# Supplementary material for: Speech prosody enhances the neural processing of syntax
Source: Commun Biol. 2024 Jun 20;7:748. doi: 10.1038/s42003-024-06444-7 (PMC11190187; doi:10.1038/s42003-024-06444-7)
Supplement: Supplementary file 2 — Supplementary Information [file 42003_2024_6444_MOESM2_ESM.docx]

# Supplementary Methods

## Speech Stimuli

During MEG acquisition, participants were presented with 7 different blocks of TED talk recording extracts of various durations, with an average of 509 seconds. Supplementary Table 1 shows how the 4 TED talks were split in the experiment.

## Null distribution assessment of cortical decoding of MEG data

The within-participant significance tests were computed with a permutation test of the AUC values. Each permutation was extracted by shuffling the original labels across all three conditions 1000 times.

The permutation test allowed us to assess if the assumption of a chance level AUC of 0.5 was valid. None of the within-participants AUC scores, across conditions and time windows, showed that the chance level of AUC = 0.5 was different from the null distribution given by the permutation. Supplementary Figure 2 shows the histograms of the results of the 1000 permutation of the first 5 participants for each condition.

## Test set results across the four binarized syntax by prosody conditions

We further unpacked the decoding results in the 4 different classes based on the presence of closing phrase boundaries (cPhBound) and on the level of prosodic boundary (PB) strength (Fig. 3).

The permutation test in the low prosodic boundary strength and cPhBound condition as well as that in the high prosodic boundary strength and no cPhBound condition did not show any significant cluster. Decoding from words with low prosodic boundary strength and no cPhBound revealed multiple clusters after word offset (Cluster 1: [0.056, 0.103]s, pVal = 0.036; Cluster 2: [0.177, 0.223]s, pVal = 0.021; Cluster 3: [0.391, 0.438]s, pVal = 0.030). Similarly, the condition with strong prosodic boundary strength and phrase boundary showed significant temporal decoding clusters that spanned across both pre- and post-word offsets, with a peak in the first 200ms post word-offset (Cluster 1: [-0.065, 0.056]s, pVal = 0.006; Cluster 2: [0.069, 0.210]s, pVal = 0.004; Cluster 3: [0.223, 0.277]s, pVal = 0.021; Cluster 4: [0.297, 0.357]s, pVal = 0.021). These latter two significant test sets together correspond to the coherent condition and therefore converge with the results presented in the paper.

*Supplementary Table 1 Information about the seven blocks of speech stimulus that were used in the experiment. The table reports lengths in seconds as well as the number of words of each snippet.*

| Block | File audio | Length (s) | #Words |
| --- | --- | --- | --- |
| 1 | DanielKahneman 2010_part1 | 516 | 1358 |
| 2 | DanielKahneman 2010_part2 | 488 | 1304 |
| 3 | JamesCameron 2010_part1 | 474 | 1397 |
| 4 | JamesCameron 2010_part2 | 519 | 1573 |
| 5 | JaneMcGonigal 2010_part1 | 549 | 1775 |
| 6 | JaneMcGonigal 2010_part2 | 631 | 2058 |
| 7 | TomWujec 2010 (entire talk) | 387 | 1124 |


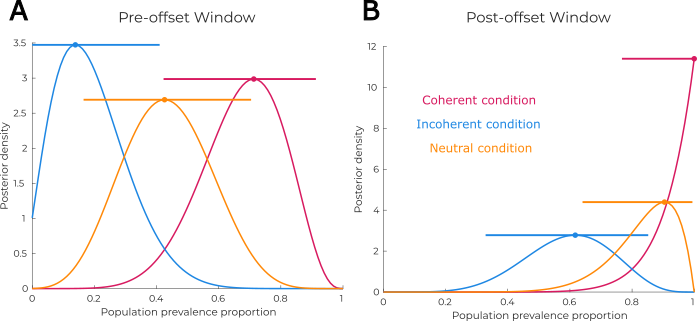


*Supplementary Figure 1: Posterior distribution of prevalence scores of the three conditions in the two-time windows before (panel A) and after token offset (panel B). Error bars at the top of the curves show the 95% highest posterior density intervals (HPDIs).*


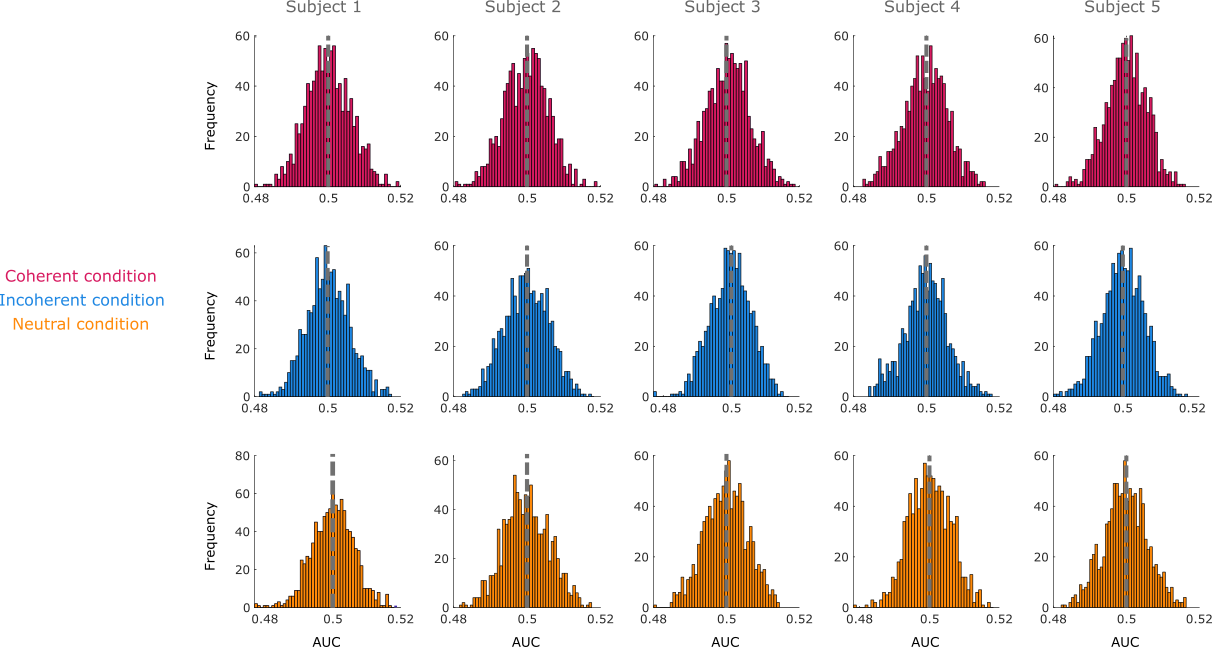


*Supplementary Figure 2: Histograms of the distribution around a chance level of 0.5 for the AUC. Columns correspond to 5 examples of subjects while rows are the results across the three different conditions. All distribution within subjects seems to follow a normal distribution*


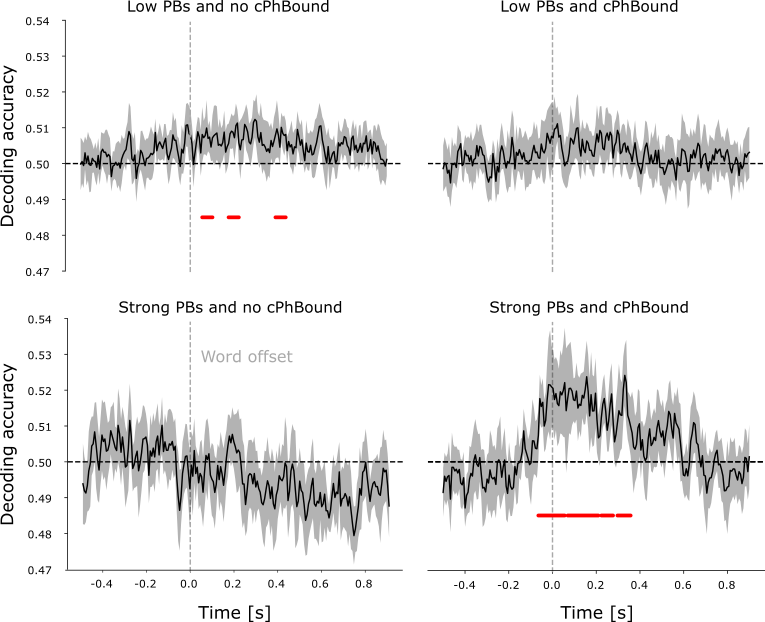


*Supplementary Figure 3: Generalization* *sets according to the four different classes of category combinations (low and high prosodic boundary strength vs presence or not of closing phrase boundaries). Red lines show significant clusters (p<0.05).*
